# A novel AI-based diagnostic model for pertussis pneumonia

**DOI:** 10.1097/MD.0000000000039457

**Published:** 2024-08-23

**Authors:** Yihong Cai, Hong Fu, Jun Yin, Yang Ding, Yanghong Hu, Hong He, Jing Huang

**Affiliations:** a Department of Pediatrics, Chongqing University Jiangjin Hospital, Chongqing, P.R. China.

**Keywords:** blood test, KNN, machine learning, pertussis, SVM, XGBoost

## Abstract

It is still very difficult to diagnose pertussis based on a doctor’s experience. Our aim is to develop a model based on machine learning algorithms combined with biochemical blood tests to diagnose pertussis. A total of 295 patients with pertussis and 295 patients with non-pertussis lower respiratory infections between January 2022 and January 2023, matched for age and gender ratio, were included in our study. Patients underwent a reverse transcription polymerase chain reaction test for pertussis and other viruses. Univariate logistic regression analysis was used to screen for clinical and blood biochemical features associated with pertussis. The optimal features and 3 machine learning algorithms including K-nearest neighbor, support vector machine, and eXtreme Gradient Boosting (XGBoost) were used to develop diagnostic models. Using univariate logistic regression analysis, 18 out of the 27 features were considered optimal features associated with pertussis The XGBoost model was significantly superior to both the support vector machine model (Delong test, *P* = .01) and the K-nearest neighbor model (Delong test, *P* = .01), with the area under the receiver operating characteristic curve of 0.96 and an accuracy of 0.923. Our diagnostic model based on blood biochemical test results at admission and XGBoost algorithm can help doctors effectively diagnose pertussis.

## 1. Introduction

Pertussis, commonly known as whooping cough, is a highly contagious respiratory disease caused by the *Bordetella pertussis* bacterium.^[1]^ Its clinical manifestations range from spasmodic coughing to complications like pneumonia, encephalopathy, respiratory failure, and even death.^[2]^ Despite widespread vaccination against pertussis in children, there has been a resurgence in its incidence in recent years, a phenomenon termed as the “pertussis renaissance.” Consequently, timely vaccination and early diagnosis and treatment become paramount to control its spread among children.

In clinical practice, the diagnosis of pertussis primarily hinges on the combination of cough and other associated symptoms.^[3]^ Studies have shown that a cough persisting for 14 days or longer is the most sensitive clinical feature of pertussis. Although specific cough characteristics (like paroxysms, the characteristic “whoop,” and post-cough vomiting) might enhance the specificity of clinical diagnosis.^[4]^ With the rise in vaccination coverage, classic cases of pertussis have been dwindling, making clinical identification challenging.^[5]^ Moreover, the inherent inaccuracies and incompleteness in symptom description, disease progression, and other features by pediatric patients further complicate the clinical diagnosis of pertussis in children. In our hospital, we have conducted biochemical blood tests on patients presenting with cough, providing several biochemical markers. However, a definitive standard for diagnosing pertussis based on these biochemical markers remains elusive.

Machine learning (ML) stands out as one of the most promising and rapidly evolving branches of Artificial Intelligence.^[6]^ It harnesses input data and selected algorithms, employing computational means to train optimal models, aiming for the best outcomes. Compared to traditional clinical diagnosis, ML leverages a more extensive data foundation, uninfluenced by subjective biases, aligning more with computational science characteristics.^[7]^ It holds a significant edge in handling vast clinical data and imaging features.^[8]^ In recent years, ML has found successful applications in various medical research endeavors, with diagnostic models significantly enhancing the speed and accuracy of diagnosing certain diseases.^[6]^ However, the potential of ML in conjunction with biochemical blood tests to aid in differentiating pertussis pneumonia remains uncharted.^[6]^

In this retrospective study, we delved into a series of cases of pertussis and other respiratory infections. We aimed to develop a model based on machine learning algorithms combined with biochemical blood tests to diagnose pertussis and assess its accuracy.^[5]^ This endeavor seeks to address the clinical challenge where, in cases where a patient’s cough persists for less than 2 weeks, doctors grapple with the uncertainty of whether the patient is suffering from pertussis.^[9]^

## 2. Materials and methods

This study was approved by the hospital’s ethics committee and informed consent was obtained from the patients’ guardians.

### 2.1. Study subjects

We retrospectively collected data on patients with pertussis treated at the Chongqing University Jiangjin Hospital from January 2022 to January 2023. Inclusion criteria were: (1) patients under 18 years of age presenting for outpatient care or at the emergency room with 1 or more apnea episodes, or paroxistic cough, whooping, or post-tussive vomiting, irrespective of the duration of cough; (2) infants with a clinical diagnosis of pertussis by a physician or respiratory symptoms and epidemiological linkage to a confirmed pertussis case were also included. Exclusion criteria included: (1) patients who did not complete sample collection by nasopharyngeal aspiration within 24 hours of admission; (2) children with other severe infectious diseases, malignant tumors, or autoimmune diseases; (3) children with incomplete clinical data.

Patients with non-pertussis lower respiratory tract infection were confirmed by PCR-fluorescent probe method within 24 hours of admission. In patients with non-pertussis lower respiratory tract infection, stratified sampling based on age and gender was used to select an equal-sized dataset as the negative samples.

### 2.2. Data analysis

Within 24 hours of admission, venous blood and respiratory secretions were collected from the patients for testing. Tests included *B pertussis* toxin antibody, complete blood count, and pathogen detection in respiratory secretions. Among the blood indicators were magnesium, α-hydroxybutyrate dehydrogenase, direct bilirubin, serum bicarbonate, total protein, and 27 other indicators. Respiratory secretion pathogen detection was conducted using a medical-standard *B pertussis* nucleic acid test kit (PCR-fluorescent probe method) to test deep nasopharyngeal aspirate 2. Diagnosis of pertussis was based on: specific IgG antibody for pertussis toxin >100,000 IU/L as positive, IgG antibody <40,000 IU/L as negative, and IgG antibody between 40,000 and 100,000 IU/L as suspected. A positive nucleic acid test confirmed the diagnosis.^[10]^

Further, we collected data on diagnosed patients, including sociodemographic variables (such as age, gender, gestational age, parents’ education and employment status, patient’s pertussis immunization status, date of symptom onset, feeding method at the time of symptom onset, number of family members, and respiratory symptoms in family members), clinical symptom information, recent medical visits, current treatment, and past pertussis vaccination history.^[10]^ All children diagnosed with pertussis were followed up to monitor the duration of their cough.

### 2.3. Feature selection based on logistic regression

Considering the potential interrelation between features and the impact of potential noise features on diagnostic classification accuracy, we first used univariate logistic regression analysis for feature selection, retaining the optimal features with *P*-values <.05 for the next phase of model establishment.^[11]^

### 2.4. Diagnostic model construction and performance evaluation

In this study, we employed support vector machine (SVM), K-nearest neighbor (KNN), and eXtreme Gradient Boosting (XGBoost), 3 different machine learning algorithms, along with the optimal features to construct a pertussis diagnostic model.^[7,12]^ We used 5-fold cross-validation and the receiver operating characteristic curve to evaluate the performance of the diagnostic model, calculating the area under the curve, accuracy, precision, recall, and F1 score for each model.^[11]^ Furthermore, we statistically analyzed the feature importance in each fold, aiming to identify core features that have a decisive impact on classification decisions.^[13]^

### 2.5. Statistical analysis

Statistical analysis was conducted using SPSS software (version 22.0). Normally distributed measurement data were expressed as mean ± standard deviation (x ± s), non-normally distributed measurement data were represented by median (first quartile, third quartile) [M(Q1, Q3)], and count data were represented by number (percentage) [n(%)].^[14]^

## 3. Results

### 3.1. Study subjects

A total of 462 pediatric patients with pertussis were included in the study. However, 167 of them were directly excluded due to insufficient laboratory items recorded in the hospital information system or obvious missing data. Ultimately, valid data from 295 cases remained. The age range of these 295 cases spanned from as young as 1 month to as old as 12 years, covering all age groups from 0 to 12 years. Among the patients, 162 were male and 133 were female.

In the cohort of non-pertussis lower respiratory tract infection patients during the same period, a total of 1173 records were obtained from individuals who completed nasopharyngeal aspirate sample collection within 24 hours and did not suffer from other severe infectious diseases, malignant tumors, or autoimmune diseases. Among them, 371 records were excluded due to insufficient or clearly missing laboratory items recorded in the hospital information system. From the remaining 802 records, a stratified sampling was conducted based on age, gender ratio, and patient symptoms to match with pediatric pertussis cases. Eventually, 295 records were selected as negative samples. The age ranged from 0 to 12 years. Among these negative samples, there were 159 males and 136 females.

During the experimental procedure, a total of 590 data points were randomly split into 70% (413 instances) for training and 30% (177 instances) for testing. This process was repeated for 5 rounds to conduct random split cross-validation. This method ensures thorough evaluation and validation of model performance across multiple iterations.

### 3.2. Feature selection

Univariate logistic regression analysis revealed that 18 out of the 27 features analyzed were considered optimal features associated with pertussis (Table 1).

**Table 1:** Feature selection of univariate Logistic regression analysis.

Laboratory indicators	Pertussis (N = 295)	Lower respiratory infections (N = 295)	*P*
Sodium (mmol/L)	129.4, 141 (136.39)	131.2, 145.1 (138.86)	<.001
Chloride (mmol/L)	83.6, 109.3 (102.89)	95.8, 112.2 (103.15)	<.001
Magnesium (mmol/L)	0.71, 1.22 (0.92)	0.78, 1.07 (0.91)	.034
α1-Microglobulin (mg/dL)	5.2, 20.4 (10.90)	6, 22.8 (12.36)	.002
Iron (μmol/L)	1.3, 23.5 (4.96)	1.8, 30.3 (10.29)	<.001
α-Hydroxybutyrate dehydrogenase (U/L)	128, 736 (231.53)	128, 263 (198.67)	<.001
Prealbumin (mg/L)	74.1, 470.8 (161.39)	56.3, 280.6 (180.52)	.036
Lactate dehydrogenase (U/L)	151, 1142 (293.00)	175, 369 (251.71)	.001
Creatinine (mg/dL)	12, 84 (29.36)	12, 60 (33.35)	.008
Uric acid (μmol/L)	106, 1371 (259.00)	139, 401 (258.19)	.007
Cystatin (mg/L)	0.59, 2.41 (1.03)	0.53, 1.5 (0.84)	<.001
Creatine kinase (U/L)	15, 2314 (123.24)	35, 774 (141.15)	<.001
Creatine kinase isoenzyme (U/L)	8, 98 (23.20)	12, 59 (25.71)	.002
Alkaline phosphatase (U/L)	96, 1656 (218.58)	139, 413 (231.13)	.015
Serum bicarbonate (mmol/L)	14, 29 (21.61)	16, 29 (22.72)	.008
Aspartate aminotransferase (U/L)	10, 373 (36.25)	14, 69 (28.58)	.001
Total protein (g/L)	52.5, 161.6 (72.47)	52.6, 85.1 (70.94)	<.001
Albumin (g/L)	36, 86.7 (45.42)	35.9, 51.4 (45.58)	<.001

### 3.3. Performance evaluation of diagnostic models

**Figure 1.**
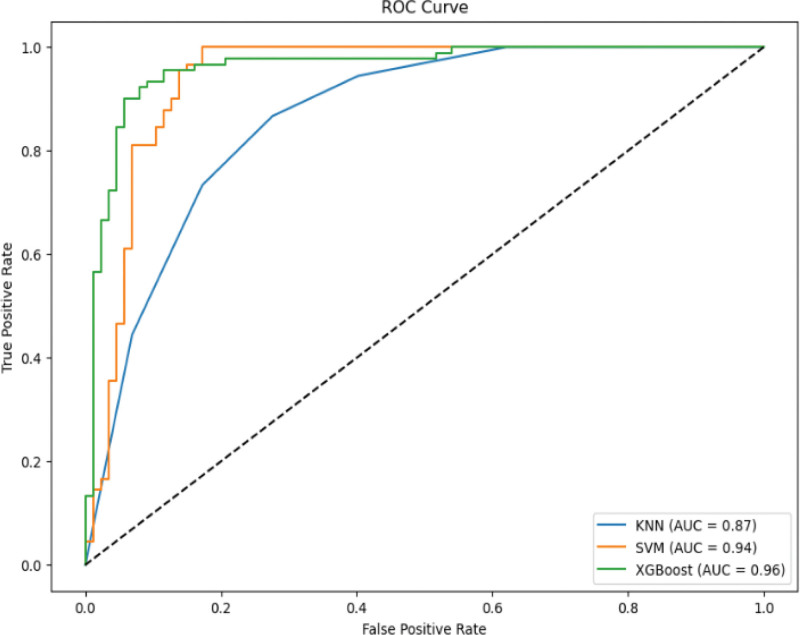
KNN, SVM, and XGBoost receiver operating characteristic (ROC) curve plots.

Experimental results indicated that the diagnostic performance of the eXtreme Gradient Boosting (XGBoost) model was significantly superior to both the SVM model (Delong test, *P* = .01) and the KNN model (Delong test, *P* = .01). Predictive results for each model can be found in Tables 2 and 3 and Figure 1.

**Table 2:** Prediction results of KNN, SVM, and XGBoost models.

Model	Accuracy	Precision	Recall	F1 score	AUC
KNN	0.758	0.724	0.840	0.777	0.87
SVM	0.858	0.862	0.893	0.877	0.94
XGBoost	0.923	0.908	0.929	0.918	0.96

**Table 3:** Confusion matrices for KNN, SVM, and XGBoost.

*KNN*	Predicted positive	Predicted negative
Actual Positive	63	24
Actual Negative	12	78
*SVM*		
Actual Positive	75	12
Actual Negative	9	81
*XGboost*		
Actual Positive	79	8
Actual Negative	6	84

## 4. Discussion

Our findings suggest that 18 blood biochemical indicators, including electrolytes, acid–base balance, liver function, heart function, and kidney function, are closely related to pertussis.^[6]^ Moreover, using these optimal features combined with 3 machine learning algorithms, we constructed diagnostic models for pertussis, with the XGBoost algorithm demonstrating the best performance.^[15]^ The model based on the XGBoost algorithm can assist clinicians in effectively distinguishing pertussis.

Humans are the only hosts for the *B pertussis* bacterium, making the general population universally susceptible. Pertussis is a vaccine-preventable disease, yet neither infection nor vaccination offers lifelong immunity.^[14]^ Clinically, we observed that although the pertussis vaccine provides individual protection, its efficacy wanes over time, especially evident in children.^[16]^ Thus, relying solely on vaccination cannot eradicate the occurrence of pertussis. Additionally, in our hospital’s clinical practice, there is a clear seasonality to pertussis outbreaks, with peaks during the summer and autumn. This aligns with other research findings.^[1]^ Summer is not only the high season for pertussis but also for other lower respiratory infections like the flu. This increases the diagnostic and care burden on hospitals. Given the two-week coughing and diagnostic period typical of pertussis cases, there is a clinical need for rapid diagnosis and triage.^[7,21]^ From our clinical interviews of the 295 children in the study, 189 had been vaccinated against pertussis within the last 2 years, accounting for 64.08%. Among these vaccinated children, 164 had mild symptoms, a significant 86.78%. This highlights that widespread vaccination has made the clinical presentation of pertussis atypical, posing a significant diagnostic challenge. Delayed diagnosis can exacerbate the condition, and given the contagious nature of pertussis, early diagnosis is crucial for controlling the source of infection, protecting susceptible children, and minimizing outbreaks.

Our results indicate that electrolyte imbalances might offer valuable clues for diagnosing pertussis.^[17]^ While these imbalances are not specific markers for the disease, their potential association with pertussis is essential for a comprehensive understanding of its clinical manifestations and physiological impacts. Firstly, these imbalances relate to inflammatory responses. Pertussis infections can trigger inflammation, affecting the body’s electrolyte regulation. Severe cases might lead to decreased serum iron levels, a response to inflammation. Monitoring serum iron concentrations can help assess the severity and activity of inflammation. Secondly, dehydration-related high serum sodium levels might be more common in pertussis patients due to persistent coughing and shortness of breath.^[18]^ While these imbalances alone are not diagnostic, they provide clinicians with crucial information about a patient’s overall health and disease progression.

Our findings also suggest that biochemical markers like lactate dehydrogenase (LDH) and prealbumin might offer new perspectives for diagnosing pertussis.^[10]^ Elevated LDH levels might reflect the presence of inflammation and cellular damage. The pathological processes of pertussis, including respiratory epithelial cell damage and inflammation, might lead to the release of LDH in tissues, causing elevated plasma LDH levels. Prealbumin, typically an indicator of nutritional status, can also be influenced by inflammation and infection. Pertussis infections can lead to anorexia, vomiting, and malnutrition, resulting in decreased prealbumin levels. While these markers are not specific for diagnosing pertussis, their variations reflect significant physiological changes during the infection, aiding in understanding its pathophysiological mechanisms.

In our study, we used the XGBoost algorithm, SVM algorithm, and KNN algorithm combined with 18 optimal blood biochemical indicators to construct a diagnostic model for pertussis.^[19,20]^ The results showed that the XGBoost model outperformed the SVM and KNN models. XGBoost, a gradient boosting algorithm, can effectively handle complex non-linear relationships and high-dimensional data. Its performance in feature selection, overfitting control, and model optimization makes it excel in constructing a diagnostic model using biochemical indicators. Moreover, XGBoost can handle imbalanced datasets, which might be useful in pertussis research, a relatively rare disease. The suboptimal performance of the SVM model might be due to the curse of dimensionality when handling high-dimensional data, requiring more feature engineering and parameter tuning.^[20]^ The KNN model’s poorest performance might be due to its high data dependency, sensitivity to noise and outliers, and the need for extensive data preprocessing to improve stability.^[18,21]^

However, our study has limitations. Being retrospective, the patient data included in the analysis is not exhaustive, and some factors might influence model construction, like pneumonia imaging features. Additionally, of the 295 children with pertussis included in the study, not all had a pure pertussis infection. One hundred seventy three had mixed infections with other pathogens, a mixed infection detection rate of 58.64%. We did not use data from children with pure pertussis infections as the basis for model suggestions and experiments, mainly considering that in clinical practice, mixed infections account for more than half, making a comprehensive approach more clinically relevant. Lastly, the artificial intelligence model we established is based on data from children in our hospital, influenced by sociological factors, and might not be applicable to all populations and regions.^[18]^

In conclusion, using blood biochemical test results at admission and the XGBoost algorithm, we successfully established a convenient and effective diagnostic model for pediatric pertussis. This model holds significance for early differential diagnosis between pediatric pertussis and other respiratory diseases in clinical practice. Especially when subjective disease descriptions are inaccurate and other testing methods are slow, it can provide timely, effective, and relatively accurate diagnostic suggestions. It can also reduce the likelihood of progression to severe cases to some extent, helping children receive treatment and be discharged sooner.

## Author contributions

**Conceptualization:** Yihong Cai, Hong He, Jing Huang.

**Data curation:** Yihong Cai, Hong Fu, Yin Jun, Yanghong Hu.

**Formal analysis:** Hong Fu, Yin Jun, Yanghong Hu.

**Investigation:** Hong Fu, Yang Ding, Jing Huang.

**Methodology:** Yihong Cai, Hong Fu, Yin Jun, Yang Ding, Hong He, Jing Huang.

**Project administration:** Jing Huang.

**Resources:** Hong He.

**Software:** Yin Jun, Jing Huang.

**Supervision:** Yang Ding.

**Validation:** Yanghong Hu.

**Visualization:** Hong Fu.

**Writing – original draft:** Yihong Cai, Hong Fu, Jing Huang.

**Writing – review & editing:** Yihong Cai, Jing Huang.
